# Nomogram-derived immune-inflammation-nutrition score could act as a novel prognostic indicator for patients with head and neck squamous cell carcinoma

**DOI:** 10.3389/fimmu.2024.1500525

**Published:** 2025-01-14

**Authors:** Wen-Yan Wang, Yue Chen, Qian Chen, Hong-Wei Sun, Nuo-Xuan Niu, Hong-Hui Li, Yu-Dan Cao, Yan-Xia Bai, Xiang Li

**Affiliations:** ^1^ Department of Otorhinolaryngology Head and Neck Surgery, The First Affiliated Hospital of Xi’an Jiaotong University, Xi’an, Shaanxi, China; ^2^ Center for Gut Microbiome Research, Med-X Institute Centre, The First Affiliated Hospital of Xi’an Jiaotong University, Xi’an, Shaanxi, China

**Keywords:** nomogram, immune-inflammation-nutrition score, prognostic, head and neck squamous cell carcinoma, indicator

## Abstract

**Aim:**

This study aims to create and validate a novel systematic immune-inflammation-nutrition (SIIN) score to provide a non-invasive and accurate prognostic tool for head and neck squamous cell carcinoma (HNSCC) patients.

**Methods:**

259 participants diagnosed with HNSCC from the First Affiliated Hospital of Xi’an Jiaotong University between 2008 and 2017 was included in this retrospective study. Patients were assigned to training (n=181) and validation (n=78) sets. A LASSO Cox regression model was employed to identify significant biomarkers for constructing a SIIN nomogram and to create SIIN score from this nomogram. The prognostic accuracy of the SIIN score was assessed by exploiting receiver operating characteristic (ROC) analysis, Kaplan-Meier survival analysis, Cox proportional hazard regression models, calibration and DCA curves.

**Results:**

The SIIN score was formulated based on six biomarkers-platelet-lymphocyte ratio (PLR), prognostic nutritional index (PNI), systemic immune-inflammation index (SII), albumin-bilirubin index (ALBI), fibrinogen (FIB) and monocyte count-identified by LASSO regression analysis. (1)The SIIN score demonstrated superior predictive value, achieving area under the ROC curve (AUC) values of 0.736 and 0.700 for 3- and 5-year OS. For recurrence-free survival (RFS), the AUC values were 0.752 for 3-year and 0.701 5-year RFS, as assessed in the training set. Validated as an independent prognostic factor in both cohorts, the SIIN score showed strong correlation with adverse clinicopathological outcomes.

**Conclusion:**

The SIIN score is a promising prognostic tool that integrates immune, inflammatory, and nutritional factors for predicting clinical outcomes in HNSCC patients. It offers enhanced predictive accuracy compared to existing markers and has the potential to guide personalized treatment strategies and clinical decision-making.

## Introduction

Encompassing a heterogeneous group of malignancies originating from the mucosal linings of the oral cavity, pharynx, larynx, and nasal cavity (1), head and neck squamous cell carcinoma (HNSCC) is the sixth most frequently occurring cancer on a global scale, characterized by a high rate of postoperative recurrence and poor survival outcomes (2). In China, there are over 130,000 new cases of HNSCC annually, resulting in approximately 70,000 deaths (3, 4). The 5-year survival rate of patients with HNSCC is 30-65%, but patients are predominantly identified at more advanced stages (III or IV), leading to only 30-50% survival rate in advanced cases (5, 6).

The aggressive nature of HNSCC, along with its high propensity for local recurrence and distant metastasis, poses a significant challenge for treatment. Despite the use of comprehensive multimodal therapies, including surgery, radiotherapy, chemotherapy, and more recently, immunotherapy, the disease remains difficult to control. Up to 50% of patients with locally advanced tumors experience recurrence after surgery, and for those whose cancer returns or spreads, the median overall survival (OS) is less than one year (7). While the absence of initial symptoms often results in delayed diagnosis, contributing to poor prognosis (8), there is an urgent need for enhanced knowledge of the disease biology and the development of more hard-hitting prognostic markers.

The majority of previous studies have established that the cancer-associated immune function, systemic inflammatory response, and nutritional status are critical indicators of prognosis in various cancers. To improve prognostic evaluations, some of these indicators have been combined into composite markers, such as platelet-lymphocyte ratio (PLR), neutrophil-lymphocyte ratio (NLR), lymphocyte-monocyte ratio (LMR), albumin-bilirubin index (ALBI) score, systemic immune-inflammation index (SII), and prognostic nutritional index (PNI) (9–15). However, these markers provide limited predictive value and do not comprehensively represent the immune, inflammatory and nutritional states of patients with HNSCC (16).

To address these limitations, we propose to develop a novel composite biomarker, the systematic immune-inflammation-nutrition (SIIN) score, which integrates immune, inflammatory, nutritional, and other clinicopathological factors to establish a practical and accurate prognostic tool. Further, we evaluated the SIIN score’s ability in the validation set to predict individualized survival, aiming to improve the accuracy of prognosis prediction and informing clinical decisions.

## Materials and methods

### Patient selection and study design

We retrospectively included 259 patients with HNSCC diagnosed and treated in the Department of Otorhinolaryngology–Head and Neck Surgery (ORL-HNS), the First Affiliated Hospital of Xi’an Jiaotong University from April 2008 to December 2017. The inclusion criteria were as follows: (1) having undergone curative resection surgery, radiotherapy, or chemoradiotherapy; (2) pathological diagnosis of primary HNSCC; and (3) availability of complete baseline clinicopathological data and follow-up information. Exclusion criteria were as follows: (1) previous diagnosis of other malignancies; (2) history of underlying health conditions about inflammatory, autoimmune, or hematological; and (3) prognostic survival of less than one month; and (4) with insufficient clinical data. The enrolled patients were arbitrarily split into a training set of 181 individuals and a validation set of 78 individuals, with a 9:4 ratio.

The study adhered to the principles outlined in the Declaration of Helsinki and was approved by the Ethics Committee of the First Affiliated Hospital of Xi’an Jiaotong University (Approval No. 2022-321). Informed consent was secured from every participant prior to their inclusion in the research. During the proofreading phase, the manuscript was reviewed and refined using GPT-4.0, a generative AI model by OpenAI, to enhance the clarity and fluency of the text.

### Data collection and variable definition

The variables collected for this study included both demographic and tumor-related factors: age, sex, smoking index, lymph node status, and tumor stage based on the 8th edition of the American Joint Committee on Cancer (AJCC) Tumor Node Metastasis (TNM) classification system. The smoking index was determined by multiplying the average daily cigarette consumption by the total duration of smoking years of the individual. Tumor staging followed the AJCC’s 8th edition TNM guidelines. Blood samples were collected within seven days before the initiation of surgery, radiotherapy (RT), or chemoradiotherapy (CRT).

Pre-treatment hematological parameters were assessed, including counts of lymphocytes, neutrophils, monocytes, and platelets, as well as levels of albumin (ALB), fibrinogen (FIB), total bilirubin (TBIL), creatinine (Cr), and alanine aminotransferase (ALT). Thirteen biomarkers related to immune function, inflammation, and nutrition were examined: lymphocyte, neutrophil, monocyte, platelet, FIB, ALB, TBIL, Cr, ALT, NLR, PLR, PNI, SII, ALBI score. The biomarkers were determined as follows: PLR = platelets/lymphocytes, NLR = neutrophils/lymphocytes, PNI = albumin (g/L) + 5 × lymphocytes, SII = platelets × neutrophils/lymphocytes, and ALBI = log_10_ total bilirubin (µmol/L) × 0.66 - albumin (g/L) × 0.085.

The primary outcome, overall survival (OS), was measured as the interval (in months) from the initial confirmed diagnosis to either death or the most recent follow-up, which occurred on December 31, 2022. Recurrence-free survival (RFS), the secondary endpoint, was measured as the period from either curative surgery or initiation of RT/CRT to first relapse last follow-up, whichever occurred first.

All data, including demographic and clinical variables, were collected from our hospital’s electronic medical records. OS and RFS outcomes were determined using multiple standardized protocols to ensure accuracy and consistency.

### Statistical analysis

All statistical analyses were conducted using IBM SPSS (version 26.0) and R software (version 4.4.1), with support from R Studio (version 2024.04.2 + 764). Constant variables were reported as means with standard deviations (SD), while categorical variables were described as frequencies with percentages. To compare clinicopathological features between the training and validation sets, various methods were used. Categorical variables were analyzed using Pearson’s Chi-square test, the Mann-Whitney U test, and Fisher’s exact test, while constant variables were assessed by Student’s t-test and the Mann-Whitney U test. The results of these comparisons are summarized in **Table 1**, with statistical significance set at p < 0.05.

**Table 1:** Comparison of clinicopathological characteristics in training and validation sets.

Variables	Training set	Validation set	Overall	P-value
	(N=181)	(N=78)	(N=259)	
Sex, N (%)				
Male	166 (91.7%)	72 (92.3%)	238 (91.9%)	0.872^a^
Female	15 (8.3%)	6 (7.7%)	21 (8.1%)	
Age, N (%)				
≥60	98 (54.1%)	46 (59.0%)	144 (55.6%)	0.473^a^
<60	83 (45.9%)	32 (41.0%)	115 (44.4%)	
Smoke index, N (%)				
≥650	70 (38.7%)	25 (32.1%)	95 (36.7%)	0.598^a^
<650	111 (61.3%)	53 (67.9%)	164 (63.3%)	
Type, N (%)				
Laryngeal cancer	143 (79.0%)	60 (76.9%)	203 (78.4%)	0.489^b^
Hypopharyngeal cancer	26 (14.4%)	15 (19.2%)	41 (15.8%)	
Others	12 (6.6%)	3 (3.8%)	15 (5.8%)	
Differentiation, N (%)				
Well	58 (32.0%)	21 (26.9%)	79 (30.5%)	0.965
Moderately	97 (53.6%)	45 (57.7%)	142 (54.8%)	
Poorly	26 (14.4%)	12 (15.4%)	38 (14.7%)	
TNM stage (AJCC, 8th), N (%)				
0/I	77 (42.5%)	25 (32.1%)	102 (39.4%)	0.162
II/III	66 (36.5%)	34 (43.6%)	100 (38.6%)	
IV	38 (20.99%)	19 (24.4%)	57 (22.0%)	
T, N (%)				
Tis	3 (1.7%)	1 (1.3%)	4 (1.5%)	0.118
T1	80 (44.2%)	27 (34.6%)	107 (41.3%)	
T2	44 (24.3%)	21 (26.9%)	65 (25.1%)	
T3	44 (24.3%)	22 (28.2%)	66 (25.5%)	
T4	10 (5.5%)	7 (9.0%)	17 (6.6%)	
N, N (%)				
N0	127 (70.2%)	49 (62.8%)	176 (68.0%)	0.310
N1	23 (12.7%)	15 (19.2%)	38 (14.7%)	
N2	31 (17.1%)	13 (16.7%)	44 (17.0%)	
N3	0 (0%)	1 (1.3%)	1 (0.4%)	
M, N (%)				
M0	175 (96.7%)	77 (98.7%)	252 (97.3%)	0.356
M1	6 (3.3%)	1 (1.3%)	7 (2.7%)	
PORT/POCRT, N (%)				
Undone	126 (69.6%)	58 (74.4%)	184 (71.0%)	0.932
Done	55 (30.4%)	20 (25.6%)	75 (29.0%)	
Lymphocyte (10^9^/L)	1.78 (0.662)	1.67 (0.718)	1.75 (0.680)	0.260^c^
Monocyte (10^9^/L)	0.429 (0.200)	0.409 (0.179)	0.423 (0.194)	0.541
Neutrophil (10^9^/L)	4.36 (2.81)	4.14 (2.42)	4.29 (2.70)	0.817
Platelet (10^9^/L)	195 (69.4)	198 (64.3)	196 (67.8)	0.616
FIB (g/L)	3.29 (0.860)	3.31 (0.960)	3.29 (0.889)	0.784
ALB (g/L)	40.1 (3.81)	39.3 (3.82)	39.9 (3.83)	0.118
TBIL (µmol/L)	11.4 (4.63)	11.3 (4.93)	11.4 (4.72)	0.716
Cr (µmol/L)	68.9 (42.2)	64.3 (15.4)	67.5 (36.3)	0.552
ALT (U/L)	24.0 (17.2)	22.4 (13.8)	23.6 (16.3)	0.503
NLR	3.21 (3.75)	3.48 (5.53)	3.29 (4.35)	0.387
PLR	130 (86.0)	148 (126)	135 (99.9)	0.148
PNI	49.0 (5.59)	47.7 (5.84)	48.6 (5.69)	0.079^c^
SII	632 (805)	723 (1430)	660 (1030)	0.249
ALBI	-2.74 (0.323)	-2.67 (0.314)	-2.72 (0.321)	0.116^c^

RT, radiotherapy; CRT, chemoradiotherapy; FIB, fibrinogen; ALB, albumin; TBIL, total bilirubin; Cr, creatinine; ALT, alanine aminotransferase; NLR, neutrophil-lymphocyte ratio; PLR, platelet-to-lymphocyte ratio; PNI, prognostic nutritional index; SII, systemic immune-inflammation index; ALBI, albumin–bilirubin.

^a^Chi-square test, ^b^Fisher exact test, ^c^Student’s t-test, others are Mann-Whitney U test.

### SIIN score construction

To identify independent prognostic biomarkers associated with immune function, nutritional status, and inflammatory response in head and neck squamous cell carcinoma patients, we conducted a comprehensive analysis using the least absolute shrinkage and selection operator (LASSO) Cox regression. This method was applied to 13 preselected variables in the training set, enabling the identification of the most statistically significant and clinically relevant predictors. At an optimal λ value of 0.08, six variables with non-zero coefficients emerged as significant: prognostic nutritional index (PNI), platelet-to-lymphocyte ratio (PLR), systemic immune-inflammation index (SII), albumin-bilirubin index (ALBI), fibrinogen (FIB), and monocyte count.

These six variables were subsequently utilized to construct a nomogram designed to estimate 1-year, 3-year, and 5-year OS for HNSCC patients. The nomogram offers a visual representation of how these biomarkers collectively influence patient outcomes, facilitating individualized prognostic assessments.

Building on this foundation, we developed the systemic immune-inflammation-nutrition (SIIN) score. This score integrates the coefficients derived from the nomogram into a single formula, allowing clinicians to calculate a comprehensive score reflecting the interplay between immune function, inflammatory response, and nutritional status.

### Validation for SIIN score

The validation of the new indicator was developed. We preliminarily explored the connections between SIIN score and other survival indices using the Mann-Whitney U test and Kruskal-Wallis test. Total points derived from the SIIN score were then incorporated into receiver operating characteristic (ROC) analysis to determine the area under the ROC curve (AUC), which was used to obtain optimal cutoff values and assess the predictive accuracy of SIIN score in both the training and validation sets. The Kaplan-Meier (K-M) curves, along with the log-rank test, and Cox proportional hazards regression models performing univariate and multivariate analyses by stepwise backward selection, were used to test the ability of the SIIN score to stratify the risk of mortality of SIIN score for OS and RFS, where statistical significance was defined as a two-sided p-value of less than 0.05. Calibration curves and decision curve analysis (DCA) were utilized to evaluate the accuracy and clinical utility of the SIIN nomogram.

## Results

### Baseline characteristics

A total of 279 HNSCC patients who participated in regular examinations and follow-up visits between April 2008 and December 2017 were excluded based on exclusion criteria, and 259 patients were included eventually, whose baseline demographic and clinical characteristics were arbitrarily assigned to either the training set (n = 181) or the validation set (n = 78), as detailed in **Table 1**. 238 (91.9%) patients of them were male, and the age of 144 (55.6%) were over 60-year-old, with no significant difference found in baseline clinicopathological parameters between training and validation sets (p > 0.05).

### Development of a nomogram for predicting a significant compositive indicator

**Figure 1:**
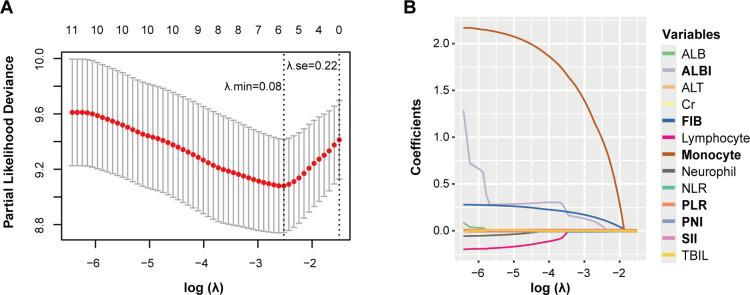
Potential prognostic factors selection using the LASSO regression model. LASSO coefficient profiles of the 13 variables constructed from the log (λ) sequence **(A)**; 1,000-fold bootstrapping resampling cross-validation for tuning variable selection in the LASSO model **(B)**. The number of variables was filtered by drawing dotted vertical lines at λ. min (left dotted line) and λ.1se (right dotted line), respectively, according to the minimum criterion. se, standard error; min, minimum.

**Figure 2:**
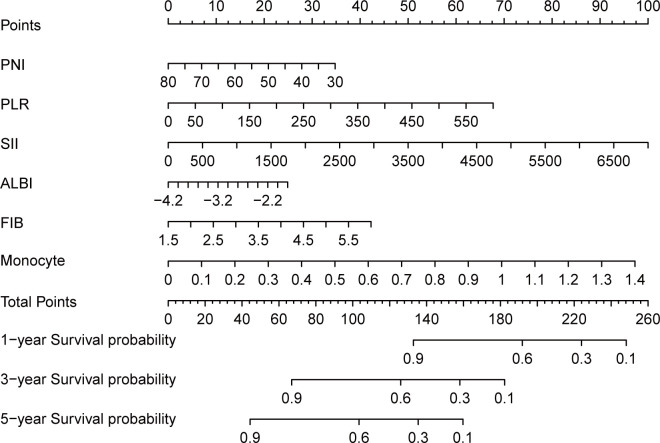
Nomogram and calculator for predicting 1, 3, 5-year overall survival of head and neck cancer patients based on LASSO regression model. For the predicting nomogram, SIIN score = - PNI × 0.115 + PLR × 0.026 + SII × 0.003 + ALBI × 0.563 + FIB × 1.233 + Monocyte × 10.034.

To identify independent prognostic biomarkers, all immune, inflammatory, and nutritional variables were incorporated in a LASSO Cox regression analysis. Using the optimal λ value corresponding to the smallest cross-validated error, this analysis identified six biomarkers - PNI, PLR, SII, ALBI, FIB, and monocyte count - with non-zero coefficients. These findings highlighted their significant association with the prognosis of HNSCC patients undergoing surgery with or without RT/CRT (**Figure 1**). The markers filtered out were put into nomogram model to construct the formula: Risk score = - PNI × 0.115 + PLR × 0.026 + SII × 0.003 + ALBI × 0.563 + FIB × 1.233 + Monocyte × 10.034 (**Figure 2**).

### Validation of SIIN score

**Figure 3:**
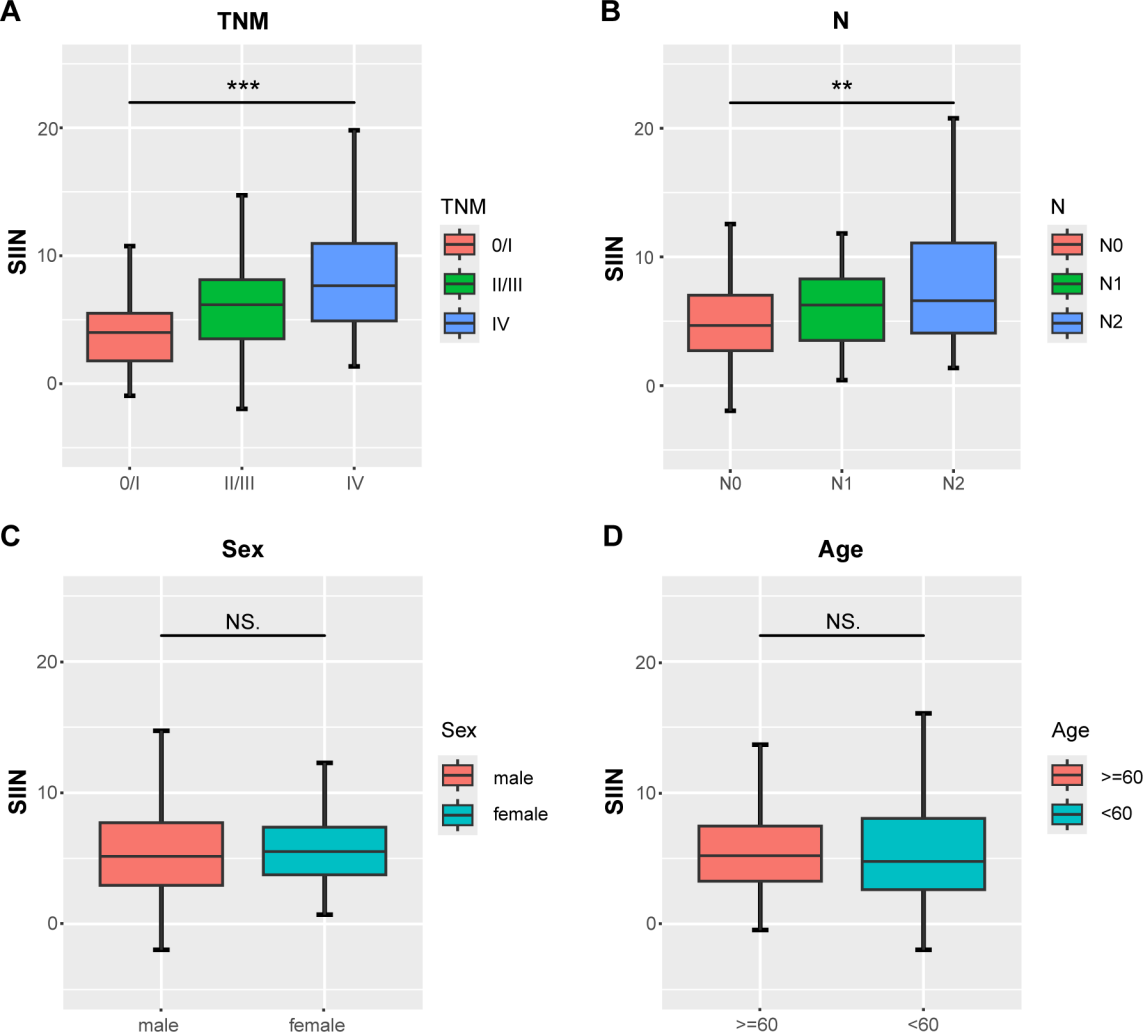
Differential analysis of the distribution of the SIIN scores in different clinicopathologic and demographic features. Comparisons between three groups such as AJCC TNM stage and lymph node status were performed using the Kruskal-Wallis test **(A, B)** and two groups including sex and age using the Mann-Whitney U test **(C, D)**. **p < 0.01; ***p < 0.001; NS, no significance.

We conducted multiple analyses to rigorously validate the prognostic capability of the SIIN score in cancer patients. Initially, we explored its correlation with key clinicopathological variables by constructing boxplots. These visualizations indicated no significant difference in SIIN scores across age and sex groups (**Figures 3A, B**). However, a clear association was observed between more advanced disease stages and higher SIIN scores. Specifically, patients with higher TNM stages and those with lymph node metastasis (N stage), consistently exhibited elevated SIIN scores (**Figures 3C, D**). This suggests that the SIIN score may reflect the degree of systemic immune, inflammatory, and nutritional alterations associated with disease progression. Consequently, the SIIN score could serve as a useful prognostic indicator in evaluating the severity of the disease and guiding treatment strategies for patients with HNSCC.

**Figure 4:**
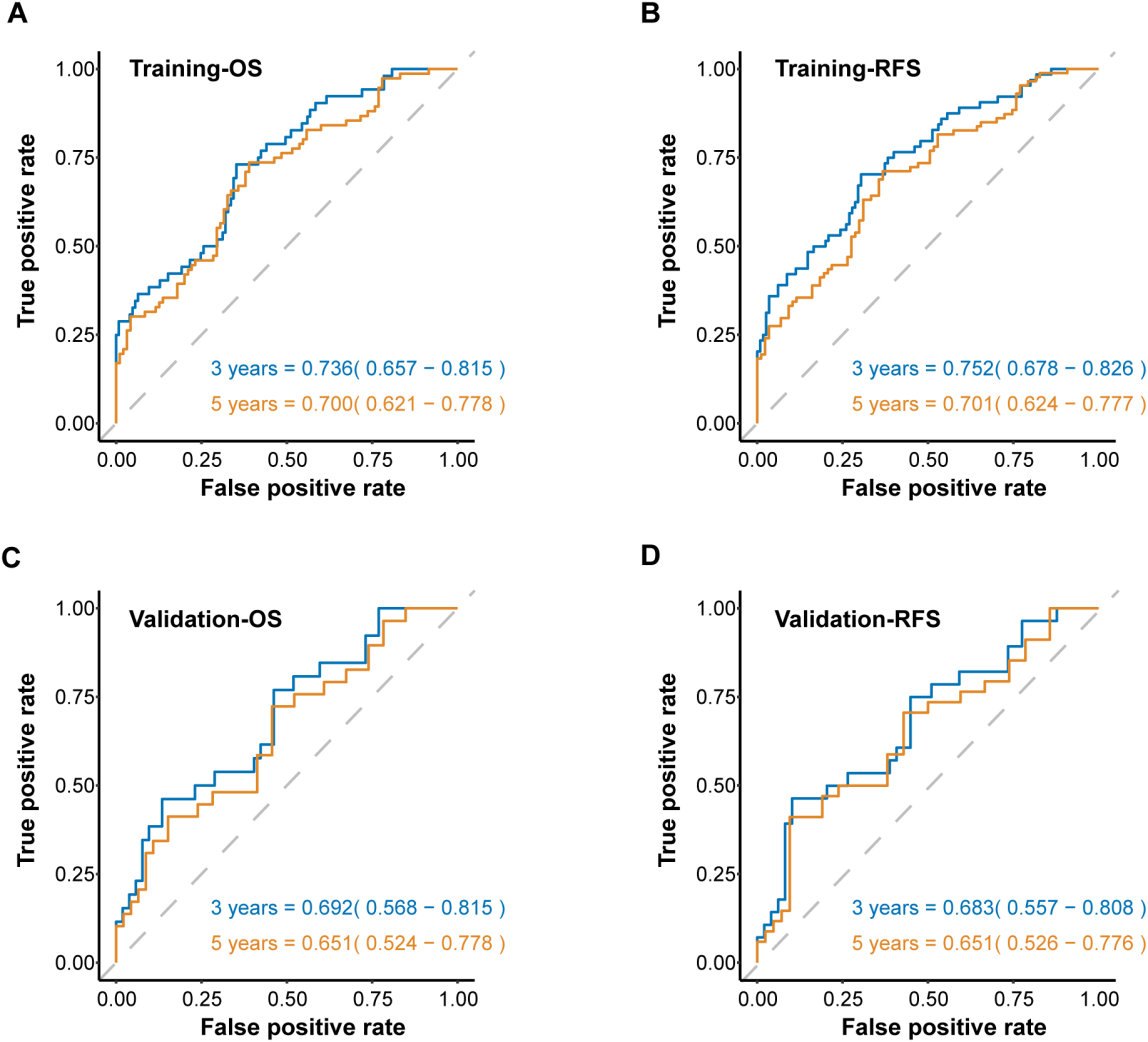
The ROC curves for predicting OS **(A)** and RFS **(B)** at 3-, and 5 years according to the SIIN score in the training set. The ROC curves for predicting OS **(C)** and RFS **(D)** at 3-, and 5 years according to the SIIN score in the validation set. ROC, receiver operating characteristic; AUC, area under the ROC curve.

To further assess the predictive strength of the SIIN score across diverse patient populations, we developed time-dependent ROC curves based on OS and RFS at different intervals. The SIIN score showed robust predictive value in the training set, with AUCs for forecasting 3- and 5-year OS of 0.736 (95% confidence interval [CI]=0.657-0.815) and 0.700 (95% CI = 0.621-0.778) (**Figure 4A**), 3- and 5-year RFS for AUCs of 0.752 (95% CI = 0.678-0.826) and 0.701 (95% CI = 0.624-0.777) (**Figure 4B**), which confirmed that the SIIN score is a reliable predictor of long-term outcomes in this population. When using time-ROC analysis in the validation set, ROC curve analysis demonstrated similar results. The AUCs for 3- and 5-year OS were 0.692 (95% CI: 0.568-0.815) and 0.651 (95% CI: 0.524-0.778) (**Figure 4C**), while the AUCs for RFS were 0.683 (95% CI: 0.557-0.808) and 0.651 (95% CI: 0.526-0.776) (**Figure 4D**). These findings suggest that the SIIN score retains its predictive accuracy in different patient subsets with HNSCC. Subsequently, to optimize clinical utility, patients were categorized into low- and high-risk groups based on a SIIN score cutoff of 4.857, derived from the ROC curve analysis (**Supplementary Figure S1**). This stratification enabled a clearer delineation of prognosis, allowing for more targeted clinical interventions for those at higher risk.

**Figure 5:**
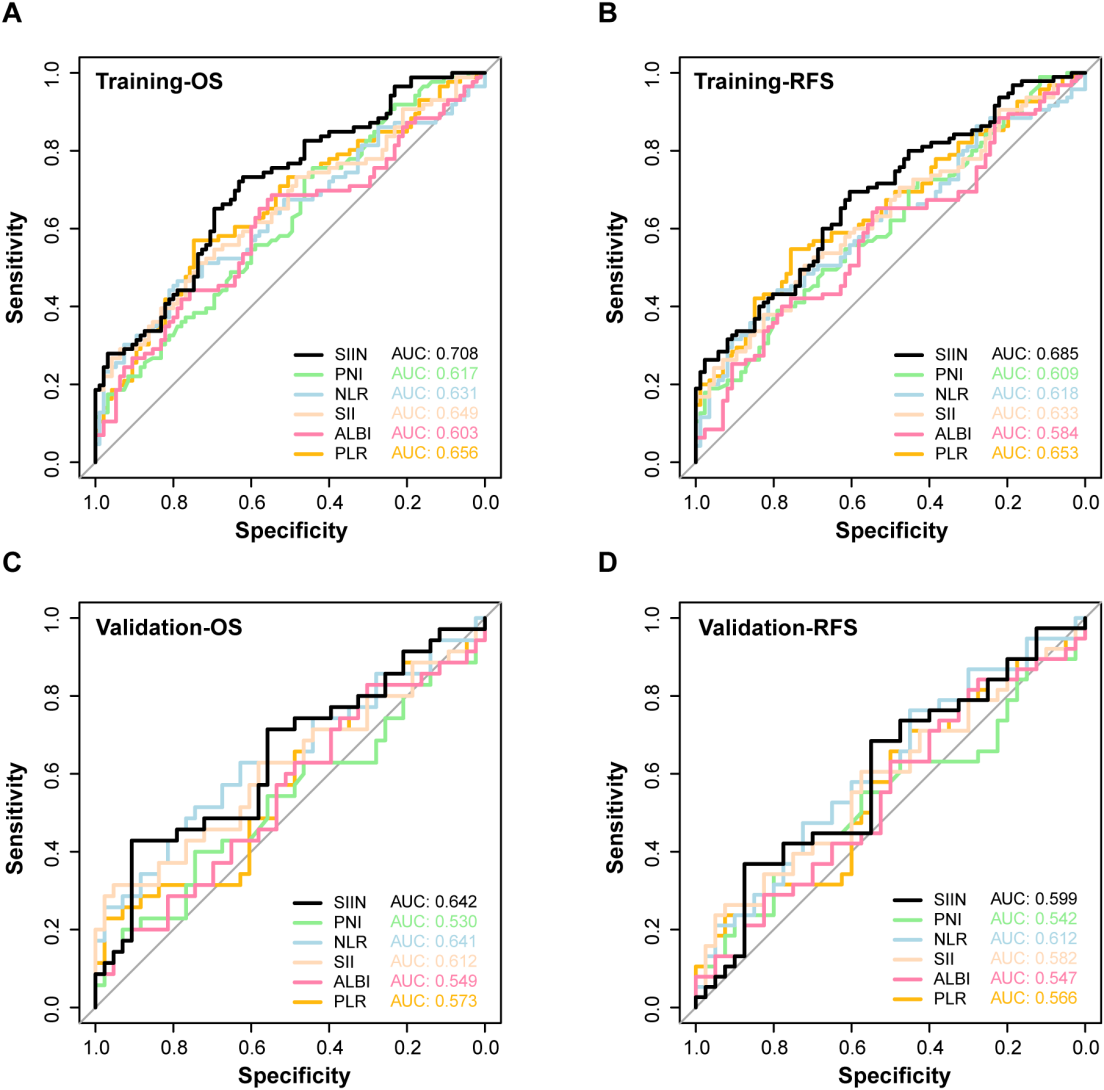
The ROC curves for predicting OS **(A)** and RFS **(B)** of SIIN and other composite markers including PNI, NLR, SII, ALBI, PLR in the training set. The ROC curves for predicting OS **(C)** and RFS **(D)** of SIIN and other composite markers including PNI, NLR, SII, ALBI, PLR in the validation set.

Next, we compared the SIIN score’s performance with other established prognostic biomarkers. The SIIN score exhibited superior predictive power, with significantly higher AUC values than those of previous comprehensive biomarkers including PNI, NLR, SII, ALBI and PLR for OS (AUC = 0.708) and RFS (AUC = 0.685) in the training set (**Figures 5A, B**), and showed consistent efficiency for OS (AUC = 0.642) and RFS (AUC = 0.599) in the validation set (**Figures 5C, D**). Meanwhile, individual blood markers including creatinine, total bilirubin, albumin, alanine aminotransferase and monocyte were verified to have lower efficiency than SIIN score across the training set (OS: AUC = 0.708; RFS: AUC = 0.685) (**Supplementary Figures S2A, B**) and the validation set (OS: AUC = 0.642; RFS: AUC = 0.599) (**Supplementary Figures S2C, D**). This highlights the SIIN score’s potential as a more precise prognostic tool in clinical practice.

**Figure 6:**
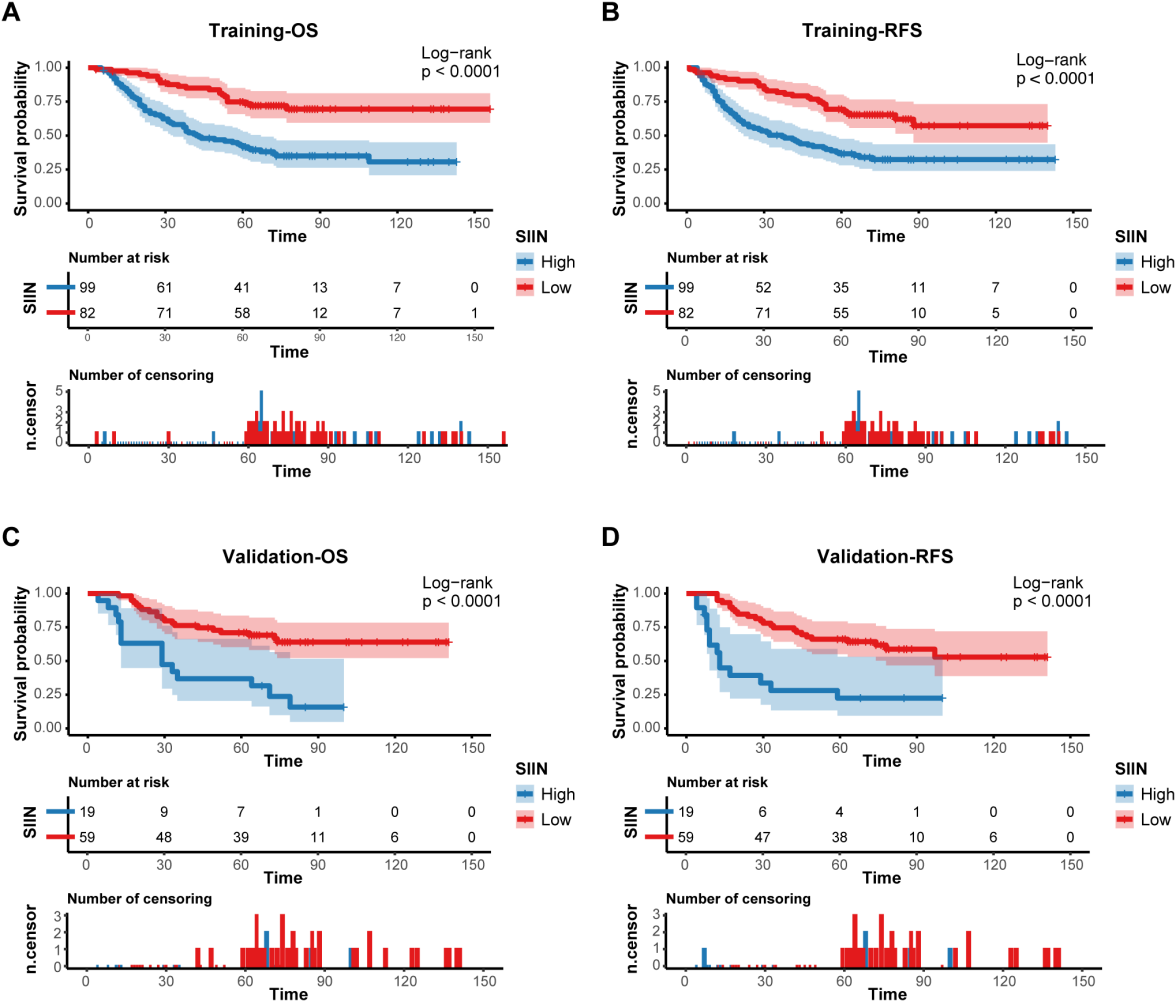
Prognostic implications of the SIIN score. Kaplan–Meier curves of OS **(A)** and RFS **(B)** for patients in the low- and high-risk groups according to the SIIN score in the training set. Kaplan–Meier curves of OS **(C)** and RFS **(D)** for patients in the low- and high-risk groups according to the SIIN score in the validation set.

Kaplan-Meier survival analysis provided further validation. Patients with higher SIIN scores had significantly poorer survival outcomes in both the training (OS: p < 0.0001; RFS: p < 0.0001) and validation sets (OS: p < 0.0001; RFS: p < 0.0001) (**Figure 6**). These results emphasize the SIIN score’s role in effectively stratifying patients based on survival risk, underscoring its value in guiding clinical decisions.

To solidify the SIIN score’s standing as an independent prognostic factor, we performed univariate and multivariate Cox regression analyses. The univariate analysis demonstrated that the SIIN score was significantly associated with OS and RFS in both the training (**Table 2**; **Supplementary Table S1**) and validation sets (**Table 3**; **Supplementary Table S2**), with p-values of less than 0.001 for all the endpoints. In the multivariate analysis, we examined clinical and pathological variables not directly linked to the SIIN score, including age, sex, smoking index, AJCC TNM stage, tumor differentiation, tumor type, and treatment modalities (RT/CRT). The multivariate results confirmed that the SIIN score remained an independent prognostic factor for OS in both the training cohort (HR 1.14, 95% CI: 1.09-1.18, p < 0.001) and the validation cohort (HR 1.04, 95% CI: 1.00-1.08, p = 0.026). Similarly, for RFS, the SIIN score continued to be a significant predictor in the training set (HR 1.13, 95% CI: 1.09-1.18, p < 0.001) and the validation set (HR 1.04, 95% CI: 1.00-1.08, p = 0.028).

**Table 2:** Univariate and multivariate cox regression analysis for OS in training set.

Variables	Univariate analysis		Multivariate analysis	
	HR (95%CI)	P value	HR (95%CI)	P value
SIIN score	1.15 (1.11-1.19)	p<.001	1.14 (1.09-1.18)	p<.001
Sex				
Female vs. Male	0.97 (0.45-2.10)	p=.940		
Age				
<60 vs. ≥60	0.73 (0.47-1.12)	p=.151	0.66 (0.42-1.04)	p=.076
Smoke index				
<650 vs. ≥650	0.67 (0.44-1.02)	p=.063	0.84 (0.53-1.33)	p=.463
TNM stage(AJCC,8th)				
0/I	Ref		Ref	
II/III	3.46 (1.95-6.14)	p<.001	3.31 (1.82-5.99)	p<.001
IV	5.66 (3.12-10.27)	p<.001	4.68 (2.33-9.41)	p<.001
Tumor differentiation				
Well Differentiated	Ref		Ref	
Moderately Differentiated	1.32 (0.80-2.19)	p=.277	0.91 (0.54-1.54)	p=.727
Poorly Differentiated	1.57 (0.82-3.04)	p=.176	1.20 (0.58-2.51)	p=.620
Tumor type				
Laryngeal cancer	Ref		Ref	
Hypopharyngeal cancer	1.37 (0.78-2.41)	p=.270	0.63 (0.32-1.22)	p=.172
Others	1.66 (0.79-3.46)	p=.179	0.83 (0.37-1.89)	p=.665
PORT/POCRT				
Done vs. Undone	1.98 (1.29-3.04)	p=.002	0.76 (0.43-1.33)	p=.335
NLR	1.11 (1.06-1.15)	p<.001		
PLR	1.01 (1.00-1.01)	p<.001		
PNI	0.91 (0.88-0.96)	p<.001		
SII	1.00 (1.00-1.00)	p<.001		
ALBI	2.99 (1.48-6.06)	p=.002		
Cr (µmol/L)	1.00 (1.00-1.01)	p=.257		
ALT (U/L)	0.99 (0.97-1.00)	p=.118		
FIB (g/L)	1.60 (1.26-2.04)	p<.001		
ALB (g/L)	0.91 (0.85-0.96)	p=.002		
TBIL (µmol/L)	0.99 (0.95-1.04)	p=.697		
Lymphocyte (10^9^/L)	0.59 (0.41-0.84)	p=.003		
Monocyte (10^9^/L)	7.63 (2.44-23.89)	p<.001		
Neutrophil (10^9^/L)	1.07 (1.02-1.12)	p=.009		
Platelet (10^9^/L)	1.00 (1.00-1.01)	p=.016		

OS, overall survival; HR, hazard ratio; CI, confidence interval; SIIN, systematic immune-inflammation-nutrition score; RT, radiotherapy; CRT, chemoradiotherapy; FIB, fibrinogen; ALB, albumin; TBIL, total bilirubin; Cr, creatinine; ALT, alanine aminotransferase; NLR, neutrophil-lymphocyte ratio; PLR, platelet-to-lymphocyte ratio; PNI, prognostic nutritional index; SII, systemic immune-inflammation index; ALBI, albumin–bilirubin; Ref, reference.

**Table 3:** Univariate and multivariate cox regression analysis for OS in validation set.

Variables	Univariate analysis		Multivariate analysis	
	HR (95%CI)	P value	HR (95%CI)	P value
SIIN score	1.05 (1.02-1.0)	p<.001	1.04 (1.00-1.08)	p=.026
Sex				
Female vs. Male	–			
Age				
<60 vs. ≥60	0.63 (0.31-1.26)	p=.189	0.51 (0.22-1.16)	p=.108
Smoke index				
<650 vs. ≥650	0.44 (0.23-0.87)	p=.018	0.61 (0.28-1.33)	p=.213
TNM stage(AJCC,8th)				
0/I	Ref		Ref	
II/III	4.66 (1.58-13.73)	p=.005	3.19 (1.01-10.10)	p=.048
IV	4.80 (1.55-14.89)	p=.007	4.26 (1.23-14.69)	p=.022
Tumor differentiation				
Well Differentiated	Ref		Ref	
Moderately Differentiated	1.48 (0.63-3.51)	p=.370	1.12 (0.46-2.74)	p=.803
Poorly Differentiated	2.50 (0.90-6.91)	p=.078	2.38 (0.73-7.69)	p=.149
Tumor type				
Laryngeal cancer	Ref			
Hypopharyngeal cancer	1.08 (0.47-2.49)	p=.865		
Others	1.52 (0.36-6.41)	p=.570		
PORT/POCRT				
Done vs. Undone	1.82 (0.91-3.62)	p=.088	0.98 (0.41-2.35)	p=.956
NLR	1.08 (1.04-1.12)	p<.001		
PLR	1.00 (1.00-1.00)	p=.005		
PNI	0.98 (0.92-1.04)	p=.552		
SII	1.00 (1.00-1.00)	p<.001		
ALBI	0.65 (0.22-1.97)	p=.447		
Cr (µmol/L)	1.02 (1.00-1.04)	p=.052		
ALT (U/L)	1.00 (0.97-1.02)	p=.704		
FIB (g/L)	1.22 (0.93-1.60)	p=.153		
ALB (g/L)	1.02 (0.93-1.12)	p=.675		
TBIL (µmol/L)	0.98 (0.91-1.05)	p=.557		
Lymphocyte (10^9^/L)	0.68 (0.41-1.15)	p=.155		
Monocyte (10^9^/L)	2.43 (0.41-14.48)	p=.331		
Neutrophil (10^9^/L)	1.23 (1.12-1.37)	p<.001		
Platelet (10^9^/L)	1.00 (0.99-1.00)	p=.798		

OS, overall survival; HR, hazard ratio; CI, confidence interval; SIIN, systematic immune-inflammation-nutrition score; RT, radiotherapy; CRT, chemoradiotherapy; FIB, fibrinogen; ALB, albumin; TBIL, total bilirubin; Cr, creatinine; ALT, alanine aminotransferase; NLR, neutrophil-lymphocyte ratio; PLR, platelet-to-lymphocyte ratio; PNI, prognostic nutritional index; SII, systemic immune-inflammation index; ALBI, albumin–bilirubin; Ref, reference.

**Figure 7:**
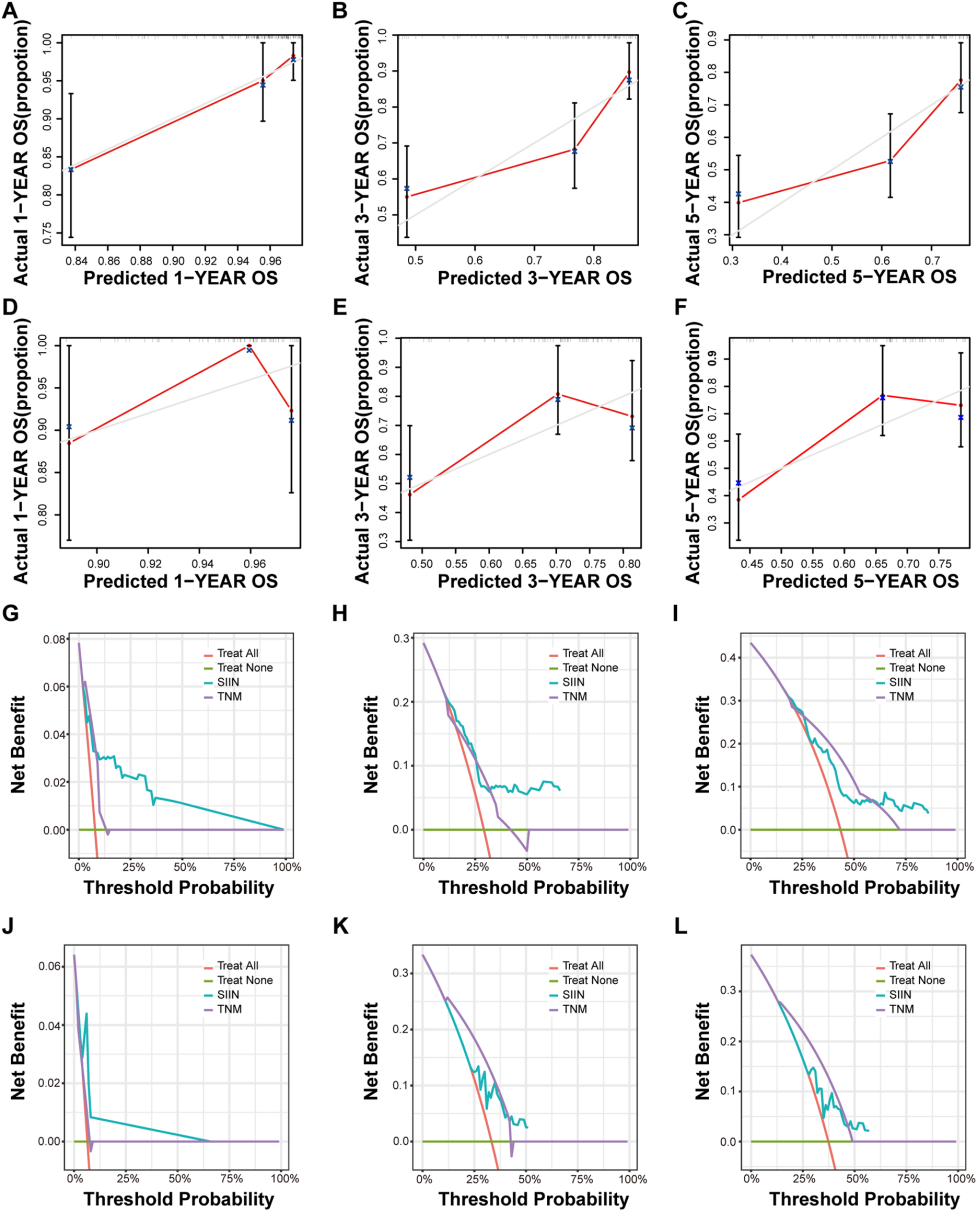
Calibration curves and DCA curves for 1, 3, 5-year OS in HNSCC patients. The calibration curves of the nomograms according to bootstrapping method between predicted and observed 1-year **(A)**, 3-year **(B)**, and 5-year **(C)** OS of patients in the training set and 1-year **(D)**, 3-year **(E)**, and 5-year **(F)** OS of patients in the validation set based on TNM stage and the SIIN score. The DCA curves for 1-year **(G)**, 3-year **(H)**, and 5-year **(I)** OS prediction of patients in the training set and 1-year **(J)**, 3-year **(K)**, and 5-year **(L)** OS prediction of patients in the validation set based on the nomograms. DCA, decision curve analysis.

Calibration curves for both training and validation sets showed strong concordance between predicted and observed outcomes, indicating excellent model calibration (**Figures 7A–F**; **Supplementary Figures S3A–F**). Furthermore, decision curve analysis (DCA) was used to assess the clinical utility of the SIIN-based nomogram model at various threshold probabilities (**Figures 7G–L**; **Supplementary Figures S3G–L**). The results demonstrated substantial net benefit across a range of threshold probabilities, confirming that the SIIN-based nomogram provides robust discrimination and valuable clinical guidance in predicting patient outcomes.

## Discussion

To the best of our knowledge, this is the first study to thoroughly evaluate the combined predictive and clinical relevance of immune, inflammatory, and nutritional parameters in determining the prognosis of HNSCC. We introduced a novel approach by integrating LASSO regression analysis with a nomogram to develop the SIIN score, which incorporates biomarkers related to immunity, inflammation, and nutrition to predict survival outcomes in HNSCC patients. This innovative methodology could serve as a valuable asset in guiding personalized therapeutic decisions, and a crucial step forward in the management of HNSCC.

Previous studies have highlighted the importance of these factors in guiding therapeutic decisions for HNSCC patients. Many have demonstrated a strong association between peripheral blood biomarkers and tumor development in HNSCC. For instance, systemic inflammatory responses, such as elevated C-reactive protein levels (17), have been found to play a crucial role in predicting patient outcomes (18), with approximately 20% of cancers globally linked to infections and inflammatory reactions (19, 20). Specifically, the role of inflammation is profound in HNSCC, influencing tumor biological behavior and patient prognosis. The dynamic interplay between systemic inflammation and immune dysregulation has even been proposed as the “seventh hallmark” of cancer, underscoring its role in initiating and perpetuating malignancies (21, 22). Inflammation within the tumor microenvironment can promote cancer cell proliferation and metastasis while suppressing effective anti-tumor immune responses. Conversely, a robust immune response can suppress or even halt tumor progression. Nutritional status is another critical factor affecting the prognosis and quality of life in cancer patients, including those with HNSCC. Malnutrition and cancer-related cachexia can lead to worse outcomes, including an increased incidence of treatment-related complications and reduced overall survival. The prognostic nutritional index (PNI), which combines serum albumin levels and lymphocyte count, is a widely used marker that reflects both nutritional and immune status (12, 13, 23). Collectively, the integration of these three parameters-immunity, inflammation, and nutrition-into a single prognostic model offers a more holistic and nuanced understanding of cancer biology with the potential to improve patient stratification and personalized treatment strategies.

Given that a single marker is inadequate for effective prognosis and risk stratification, our study emphasizes the importance of integrating multiple biomarkers. We collected data on immune, inflammatory, and nutritional biomarkers from the peripheral blood of HNSCC patients and identified six factors independently associated with patient survival using LASSO regression analysis: platelet-lymphocyte ratio (PLR), prognostic nutritional index (PNI), systemic immune-inflammation Index (SII), albumin-bilirubin (ALBI) score, fibrinogen (FIB), and monocyte count. These biomarkers were then combined in a nomogram to create the SIIN score model, which has been validated as a reliable tool for prognostic stratification in both training and validation sets.

The SIIN score incorporates six biomarkers - PNI, PLR, SII, ALBI, FIB and monocyte - that reflect the immune response, inflammation reaction, and nutrition status. The prognostic significance of these markers has been validated in various cancers. For instance, PNI, which can reflect the chronic inflammation, immune status, and nutritional status of the patients with cancer (24), is regarded as a promising prognostic factor for HNSCC patients (25). Similarly, PLR, calculated by combing platelet and lymphocyte counts, has been linked to tumorigenesis, invasiveness, and poor prognosis in many cancer types (9, 26, 27). Research by Takenaka et al. demonstrated that PLR could also serve as a valuable important prognostic factor for patients with HNSCC (15). SII, which includes peripheral lymphocyte, platelet, and neutrophil counts, is another novel biomarker that effectively estimates a patient’s immune response and inflammatory status. High SII levels, associated with cancer progression and reduced survival, are thought to result from interactions between these three cell types: neutrophilia and thrombocythemia may contribute to cancer progression, while lymphocytes exert an antitumor effect by suppressing tumor proliferation and inducing cytotoxic cell death (11, 28–32). The ALBI score, originally proposed by Johnson et al. in 2015 as a measure of liver function, has also been validated in our study as a nutrition-related prognostic factor for HNSCC (10). Furthermore, elevated monocyte has been linked to poorer outcomes across various cancers by promoting tumor growth and angiogenesis while suppressing antitumor immune responses (33). Fibrinogen is a blood protein involved in blood clotting and inflammation. Elevated levels of fibrinogen are associated with a hypercoagulable state, which can contribute to tumor development and metastasis by promoting angiogenesis (the formation of new blood vessels) and facilitating tumor spread (34–36). All biomarkers in the SIIN score can be easily obtained from routine preoperative blood tests, providing a practical and effective tool for clinical use. In conclusion, the SIIN-nomogram developed by integrating these factors can effectively predict malnutrition, immunosuppression, inflammatory activity, and tumor progression in HNSCC patients.

However, the present study should be viewed in light of several limitations: (1) As a retrospective analysis with a relatively small sample size, the findings may lack generalizability, particularly since all participants were Chinese. Future multi-center studies involving more diverse populations are essential to confirm the prognostic value of the SIIN score; (2) The study’s reliance on blood samples collected only at baseline restricts insights into the long-term dynamics of these biomarkers during treatment. Longitudinal studies are needed to better understand how these biomarkers evolve over time and their relationship to patient outcomes; (3) Additional research at genomic, transcriptomic, and proteomic levels is crucial to clarify the biological and prognostic differences among patients with HNSCC. Such investigations could enhance the precision of prognostic tools and deepen our understanding of the disease; (4) The development of accessible and user-friendly software tools is necessary to streamline the calculation and application of the SIIN score in clinical settings. These advancements would facilitate its integration into routine practice and improve its utility for personalized patient management.

## Conclusion

In summary, the development of the SIIN score combining immune, inflammatory, and nutritional markers represents a significant advance in the field of HNSCC prognosis. By capturing the complex interactions among these critical biological processes, this tool has the potential to refine prognostic predictions and support more tailored treatment decisions to ultimately improve patient outcomes. Further studies are warranted to validate the SIIN score in larger, diverse cohorts and to explore its utility in clinical practice, particularly in emerging immunotherapies and targeted treatments for HNSCC.

## Data Availability

The original contributions presented in the study are included in the article/**Supplementary Material**, Further inquiries can be directed to the corresponding author/s.

## Supplementary material

Supplementary Figure 1 The ROC curves for predicting OS in the training set according to the SIIN score. ROC, receiver operating characteristic; AUC, area under the ROC curve.

Supplementary Figure 2 The ROC curves for predicting OS **(A)** and RFS **(B)** of SIIN and other individual factors including Cr, TBIL, ALB, ALT, Mon in the training set. The ROC curves for predicting OS **(C)** and RFS **(D)** of SIIN and other individual factors including Cr, TBIL, ALB, ALT, Mon in the validation set. Mon, monocyte. ROC, receiver operating characteristic; AUC, area under the ROC curve.

Supplementary Figure 3 Calibration curves and DCA curves for 1, 3, 5-year RFS in HNSCC patients. The calibration curves of the nomograms between predicted and observed 1-year **(A)**, 3-year **(B)**, and 5-year **(C)** RFS of patients in the training set and 1-year **(D)**, 3-year **(E)**, and 5-year **(F)** RFS of patients in the validation set. The DCA curves for 1-year **(G)**, 3-year **(H)**, and 5-year **(I)** RFS prediction of patients in the training set and 1-year **(J)**, 3-year **(K)**, and 5-year **(L)** RFS prediction in the validation set based on the nomograms. DCA, decision curve analysis.
