# Supplementary figures and images for: Nomogram-derived immune-inflammation-nutrition score could act as a novel prognostic indicator for patients with head and neck squamous cell carcinoma
Source: Front Immunol. 2025 Jan 14;15:1500525. doi: 10.3389/fimmu.2024.1500525 (PMC11772279; doi:10.3389/fimmu.2024.1500525)

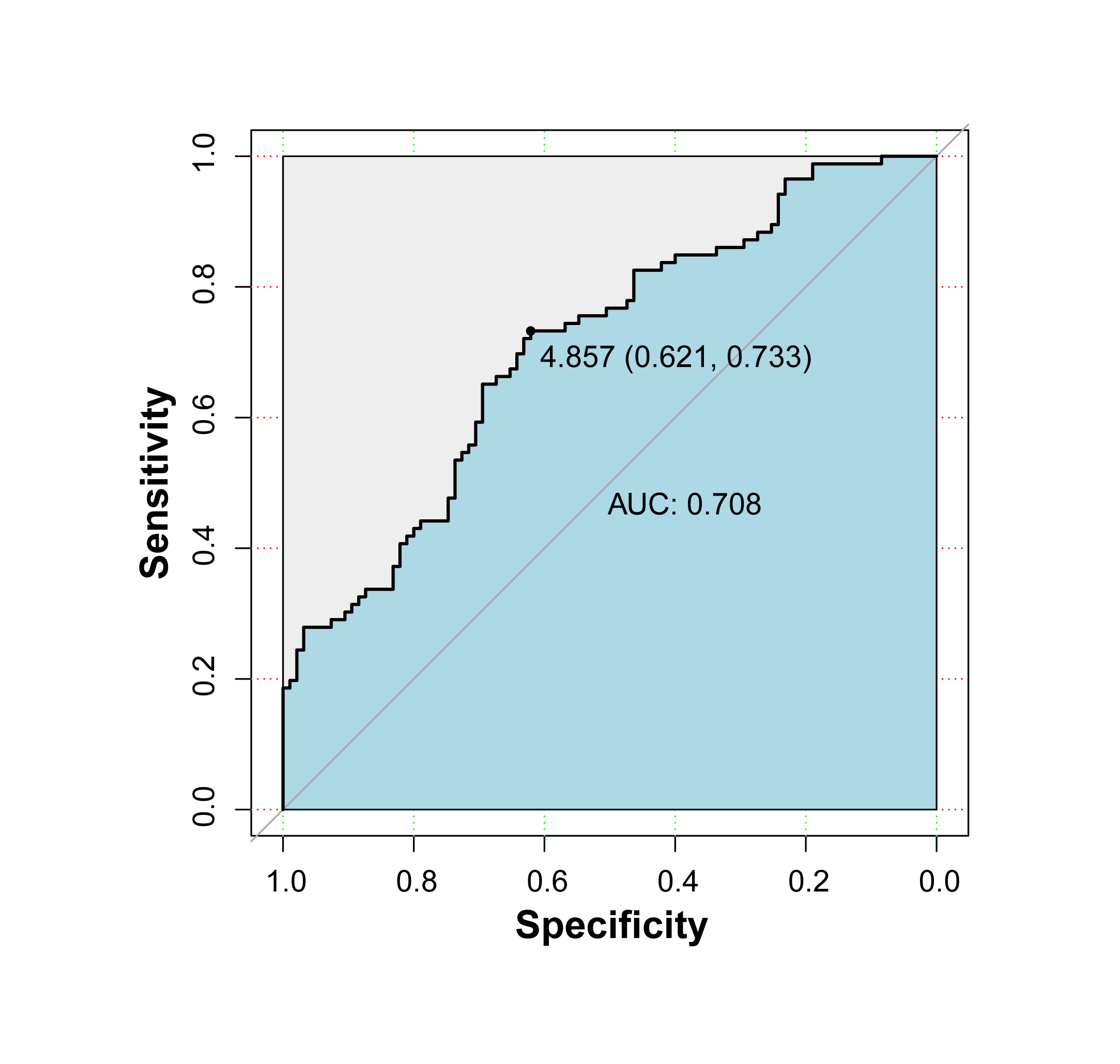

Supplement: Supplementary Figure 1 — The ROC curves for predicting OS in the training set according to the SIIN score. ROC, receiver operating characteristic; AUC, area under the ROC curve. [file Image1.tif]

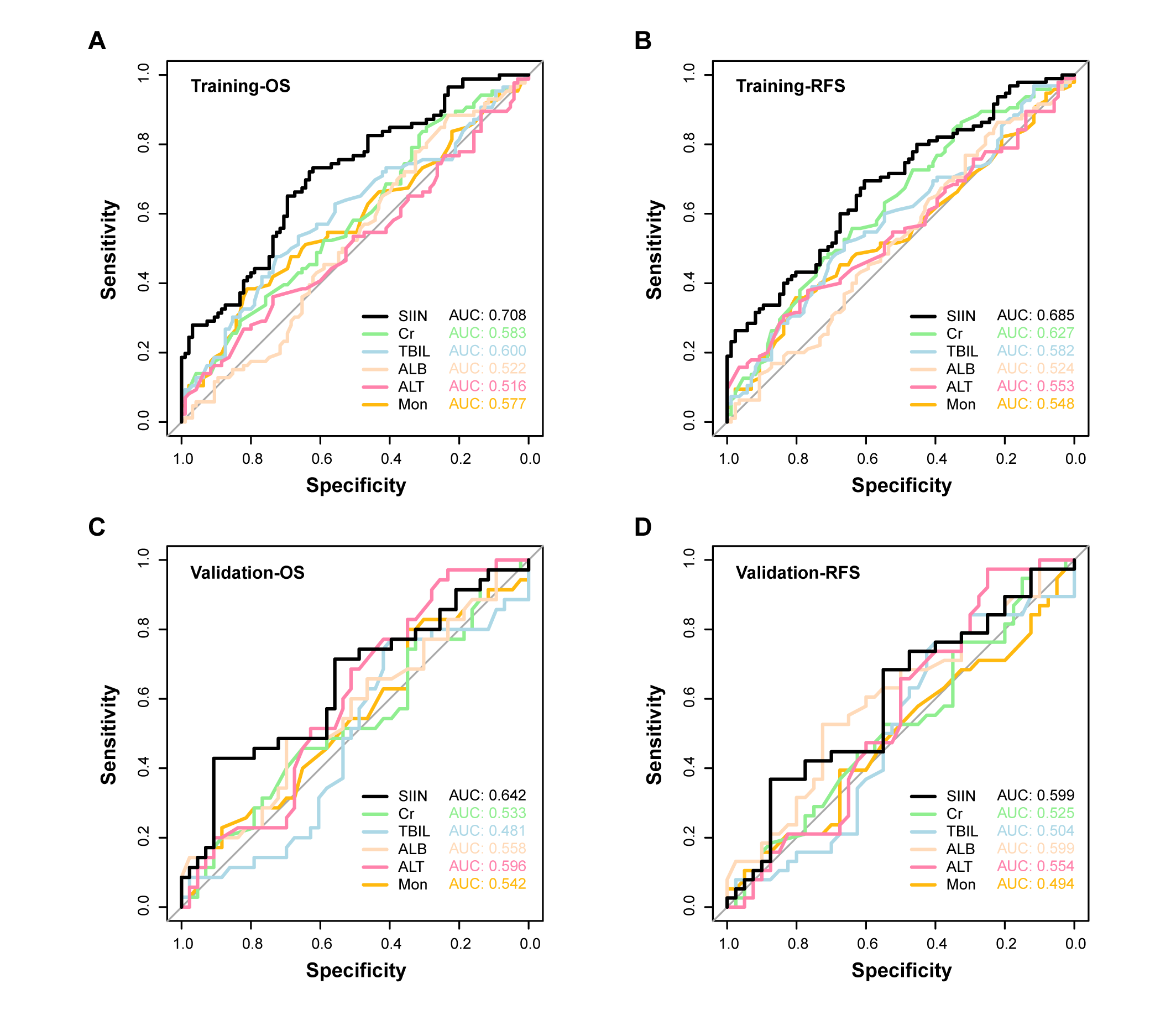

Supplement: Supplementary Figure 2 — The ROC curves for predicting OS (A) and RFS (B) of SIIN and other individual factors including Cr, TBIL, ALB, ALT, Mon in the training set. The ROC curves for predicting OS (C) and RFS (D) of SIIN and other individual factors including Cr, TBIL, ALB, ALT, Mon in the validation set. Mon, monocyte. ROC, receiver operating characteristic; AUC, area under the ROC curve. [file Image2.tif]

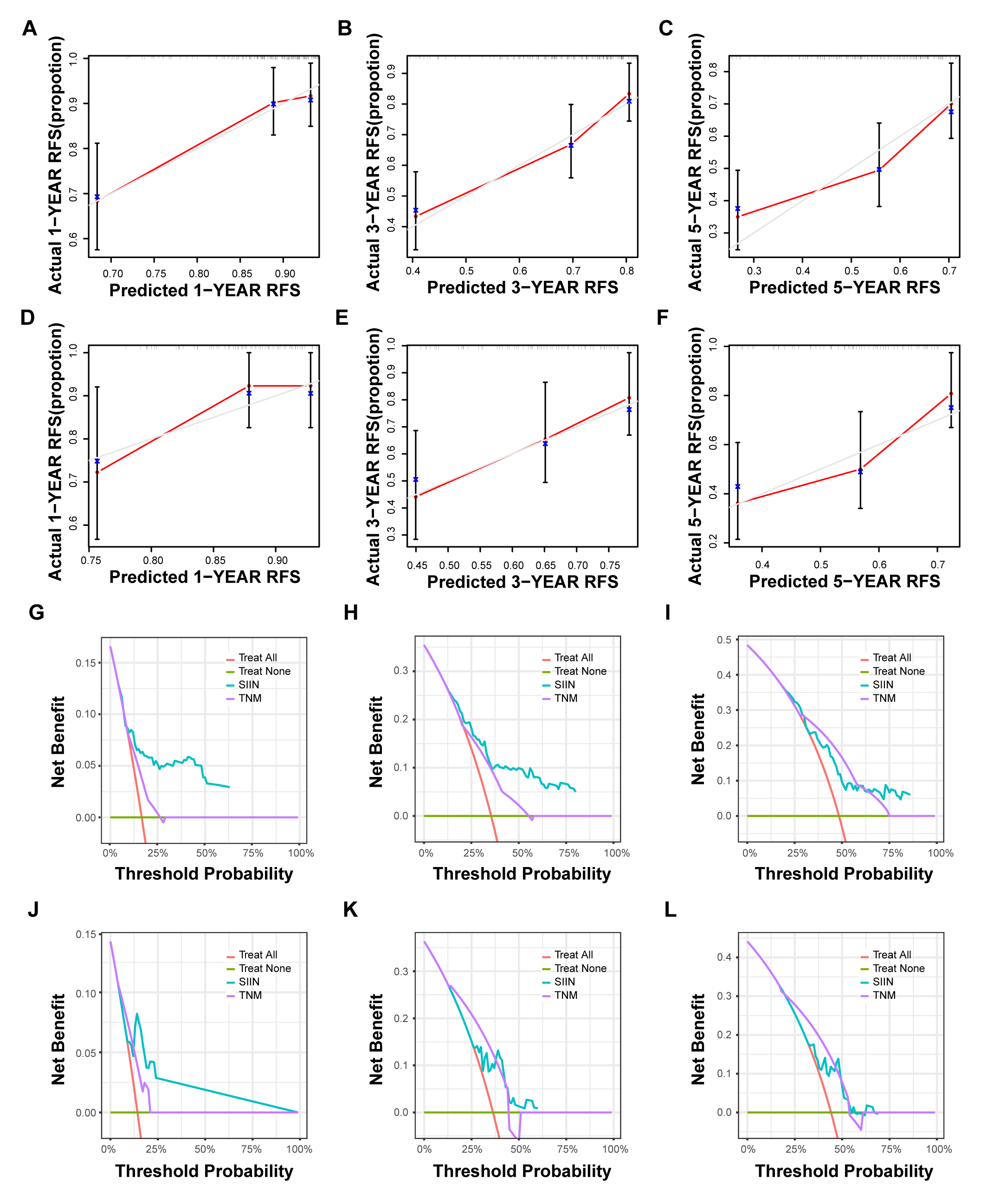

Supplement: Supplementary Figure 3 — Calibration curves and DCA curves for 1, 3, 5-year RFS in HNSCC patients. The calibration curves of the nomograms between predicted and observed 1-year (A), 3-year (B), and 5-year (C) RFS of patients in the training set and 1-year (D), 3-year (E), and 5-year (F) RFS of patients in the validation set. The DCA curves for 1-year (G), 3-year (H), and 5-year (I) RFS prediction of patients in the training set and 1-year (J), 3-year (K), and 5-year (L) RFS prediction in the validation set based on the nomograms. DCA, decision curve analysis. [file Image3.tif]
