# Supplementary material for: Nomogram-derived immune-inflammation-nutrition score could act as a novel prognostic indicator for patients with head and neck squamous cell carcinoma
Source: Front Immunol. 2025 Jan 14;15:1500525. doi: 10.3389/fimmu.2024.1500525 (PMC11772279; doi:10.3389/fimmu.2024.1500525)
Supplement: Supplementary file 4 [file Table1.docx]

**Supplementary Table 1 Univariate and Multivariate Cox Regression analysis for RFS in training set**

| **Variables** | **Univariate analysis** | | **Multivariate analysis** | |
| --- | --- | --- | --- | --- |
|  | **HR(95%CI)** | **P value** | **HR(95%CI)** | **P value** |
| SIIN score | 1.16 (1.12-1.20) | p<.001 | 1.13 (1.09-1.18) | p<.001 |
| Sex |  |  |  |  |
| Female vs. Male | 1.33 (0.69-2.56) | p=.398 |  |  |
| Age |  |  |  |  |
| <60 vs. ≥60 | 0.69 (0.46-1.05) | p=.082 | 0.72 (0.47-1.11) | p=.138 |
| Smoke index |  |  |  |  |
| <650 vs. ≥650 | 0.65 (0.43-0.97) | p=.035 | 0.80 (0.52-1.24) | p=.322 |
| TNM stage(AJCC,8th) |  |  |  |  |
| 0/I | Ref |  | Ref |  |
| II/III | 2.54 (1.54-4.20) | p<.001 | 2.07 (1.22-3.49) | p=.007 |
| IV | 4.09 (2.39-6.99) | p<.001 | 2.25 (1.19-4.23) | p=.012 |
| Tumor differentiation |  |  |  |  |
| Well Differentiated | Ref |  |  |  |
| Moderately Differentiated | 1.01 (0.64-1.60) | p=.953 |  |  |
| Poorly Differentiated | 1.32 (0.72-2.43) | p=.375 |  |  |
| Tumor type |  |  |  |  |
| Laryngeal cancer | Ref |  | Ref |  |
| Hypopharyngeal cancer | 1.58 (0.94-2.65) | p=.086 | 0.66 (0.36-1.23) | p=.190 |
| Others | 1.47 (0.71-3.06) | p=.303 | 0.84 (0.38-1.86) | p=.662 |
| RT/CRT |  |  |  |  |
| Done vs. Undone | 2.53 (1.68-3.81) | p<.001 | 1.51 (0.88-2.59) | p=.134 |
| NLR | 1.12 (1.07-1.17) | p<.001 |  |  |
| PLR | 1.01 (1.01-1.01) | p<.001 |  |  |
| PNI | 0.91 (0.88-0.95) | p<.001 |  |  |
| SII | 1.00 (1.00-1.00) | p<.001 |  |  |
| ALBI | 2.46 (1.25-4.83) | p=.009 |  |  |
| Cr (µmol/L) | 1.00 (1.00-1.01) | p=.659 |  |  |
| ALT (U/L) | 0.98 (0.97-1.00) | p=.039 |  |  |
| FIB (g/L) | 1.52 (1.21-1.91) | p<.001 |  |  |
| ALB (g/L) | 0.92 (0.87-0.97) | p=.005 |  |  |
| TBIL (µmol/L) | 0.99 (0.95-1.03) | p=.585 |  |  |
| Lymphocyte (10^9^/L) | 0.54 (0.38-0.77) | p<.001 |  |  |
| Monocyte (10^9^/L) | 4.27 (1.43-12.78) | p=.009 |  |  |
| Neutrophil (10^9^/L) | 1.07 (1.02-1.12) | p=.006 |  |  |
| Platelet (10^9^/L) | 1.00 (1.00-1.01) | p=.006 |  |  |

RFS, recurrence-free survival; HR, hazard ratio; CI, confidence interval; SIIN, systematic immune-inflammation-nutrition score; RT, radiotherapy; CRT, chemoradiotherapy; FIB, fibrinogen; ALB, albumin; TBIL, total bilirubin; Cr, creatinine; ALT, alanine aminotransferase; NLR, neutrophil-lymphocyte ratio; PLR, platelet-to-lymphocyte ratio; PNI, prognostic nutritional index; SII, systemic immune-inflammation index; ALBI, albumin–bilirubin; Ref, reference.
