# Supplementary material for: Nomogram-derived immune-inflammation-nutrition score could act as a novel prognostic indicator for patients with head and neck squamous cell carcinoma
Source: Front Immunol. 2025 Jan 14;15:1500525. doi: 10.3389/fimmu.2024.1500525 (PMC11772279; doi:10.3389/fimmu.2024.1500525)
Supplement: Supplementary file 5 [file Table2.docx]

**Supplementary Table 2 Univariate and Multivariate Cox Regression analysis for RFS in validation set**

| **Variables** | **Univariate analysis** | | **Multivariate analysis** | |
| --- | --- | --- | --- | --- |
|  | **HR(95%CI)** | **P value** | **HR(95%CI)** | **P value** |
| SIIN score | 1.06 (1.03-1.09) | p<.001 | 1.04 (1.00-1.08) | p=.028 |
| Sex |  |  |  |  |
| Female vs. Male | 0.24 (0.03-1.72) | p=.154 | 0.39 (0.05-3.18) | p=.381 |
| Age |  |  |  |  |
| <60 vs. ≥60 | 0.79 (0.41-1.52) | p=.484 |  |  |
| Smoke index |  |  |  |  |
| <650 vs. ≥650 | 0.49 (0.26-0.94) | p=.032 | 0.59 (0.28-1.22) | p=.152 |
| TNM stage(AJCC,8th) |  |  |  |  |
| 0/I | Ref |  | Ref |  |
| II/III | 3.91(1.45-10.50) | p=.007 | 2.50 (0.88-7.14) | p=.086 |
| IV | 4.67(1.68-12.98) | p=.003 | 2.55 (0.81-8.03) | p=.109 |
| Tumor differentiation |  |  |  |  |
| Well Differentiated | Ref |  | Ref |  |
| Moderately Differentiated | 1.46 (0.65-3.28) | p=.361 | 1.15 (0.51-2.62) | p=.731 |
| Poorly Differentiated | 2.41 (0.90-6.47) | p=.080 | 1.77 (0.58-5.39) | p=.316 |
| Tumor type |  |  |  |  |
| Laryngeal cancer | Ref |  |  |  |
| Hypopharyngeal cancer | 1.08 (0.49-2.39) | p=.842 |  |  |
| Others | 1.54 (0.36-6.48) | p=.558 |  |  |
| RT/CRT |  |  |  |  |
| Done vs. Undone | 2.50 (1.28-4.86) | p=.007 | 1.54 (0.65-3.65) | p=.330 |
| NLR | 1.10 (1.05-1.14) | p<.001 |  |  |
| PLR | 1.00 (1.00-1.01) | p<.001 |  |  |
| PNI | 0.97 (0.91-1.03) | p=.363 |  |  |
| SII | 1.00 (1.00-1.00) | p<.001 |  |  |
| ALBI | 0.64 (0.21-1.93) | p=.430 |  |  |
| Cr (µmol/L) | 1.01 (0.99-1.03) | p=.262 |  |  |
| ALT (U/L) | 0.99 (0.97-1.02) | p=.517 |  |  |
| FIB (g/L) | 1.16 (0.88-1.53) | p=.290 |  |  |
| ALB (g/L) | 1.01 (0.92-1.11) | p=.805 |  |  |
| TBIL (µmol/L) | 0.96 (0.89-1.03) | p=.288 |  |  |
| Lymphocyte (10^9^/L) | 0.64 (0.38-1.08) | p=.094 |  |  |
| Monocyte (10^9^/L) | 1.71(0.27-10.69) | p=.565 |  |  |
| Neutrophil (10^9^/L) | 1.23 (1.07-1.42) | p=.004 |  |  |
| Platelet (10^9^/L) | 1.00 (0.99-1.00) | p=.719 |  |  |

RFS, recurrence-free survival; HR, hazard ratio; CI, confidence interval; SIIN, systematic immune-inflammation-nutrition score; RT, radiotherapy; CRT, chemoradiotherapy; FIB, fibrinogen; ALB, albumin; TBIL, total bilirubin; Cr, creatinine; ALT, alanine aminotransferase; NLR, neutrophil-lymphocyte ratio; PLR, platelet-to-lymphocyte ratio; PNI, prognostic nutritional index; SII, systemic immune-inflammation index; ALBI, albumin–bilirubin; Ref, reference.
